# HSDFinder: A BLAST-Based Strategy for Identifying Highly Similar Duplicated Genes in Eukaryotic Genomes

**DOI:** 10.3389/fbinf.2021.803176

**Published:** 2021-12-16

**Authors:** Xi Zhang, Yining Hu, David Roy Smith

**Affiliations:** ^1^ Department of Biochemistry and Molecular Biology, Dalhousie University, Halifax, NS, Canada; ^2^ Institute for Comparative Genomics, Dalhousie University, Halifax, NS, Canada; ^3^ Department of Computer Science, Western University, London, ON, Canada; ^4^ Department of Biology, Western University, London, ON, Canada

**Keywords:** comparative genomics, genome duplication, genome evolution, gene duplication, paralogous genes

## Abstract

Gene duplication is an important evolutionary mechanism capable of providing new genetic material for adaptive and nonadaptive evolution. However, bioinformatics tools for identifying duplicate genes are often limited to the detection of paralogs in multiple species or to specific types of gene duplicates, such as retrocopies. Here, we present a user-friendly, BLAST-based web tool, called HSDFinder, which can identify, annotate, categorize, and visualize highly similar duplicate genes (HSDs) in eukaryotic nuclear genomes. HSDFinder includes an online heatmap plotting option, allowing users to compare HSDs among different species and visualize the results in different Kyoto Encyclopedia of Genes and Genomes (KEGG) pathway functional categories. The external software requirements are BLAST, InterProScan, and KEGG. The utility of HSDFinder was tested on various model eukaryotic species, including *Chlamydomonas reinhardtii*, *Arabidopsis thaliana*, *Oryza sativa*, and *Zea mays* as well as the psychrophilic green alga *Chlamydomonas* sp. UWO241, and was proven to be a practical and accurate tool for gene duplication analyses. The web tool is free to use at http://hsdfinder.com. Documentation and tutorials can be found via the GitHub: https://github.com/zx0223winner/HSDFinder.

## Introduction

Gene duplication is a near-ubiquitous phenomenon throughout the eukaryotic tree of life (Ohno, 1970). Sometimes it is beneficial, providing the raw genetic material for the acquisition of new functions (Conant and Wolfe, 2008). Other times it is deleterious. For example, the expression of near-identical genes can be disadvantageous in certain situations (Conrad and Antonarakis, 2007), which is perhaps why their presence is quite rare in eukaryotic genomes (Kubiak and Makałowska, 2017). Nevertheless, the maintenance of highly similar duplicate genes (HSDs) is possible if, for instance, the duplicates in question are in high demand, such as those encoding rRNAs or histones (Zhang, 2003). The presence of HSDs in genomes can also reflect recent duplication events, possibly representing duplicates that are potentially drifting to extinction (Conant and Wagner, 2002).

Duplicated genes formed and retained by various mechanisms and models have been widely discussed (Koonin, 2005; Innan and Kondrashov, 2010), and it is generally accepted that neutral processes are the primary drivers of duplicate gene evolution, particularly their appearance and loss from genomes through genetic drift (Nei and Roychoudhury, 1973; Li, 1980; Lynch, 2007; Brunet and Doolittle, 2018). However, there are various theories for how duplicate genes can be fixed by adaptive evolution, including the gene dosage hypothesis (Qian and Zhang, 2008), the “Escape from adaptive conflict” model (Des Marais and Rausher, 2008) and Ohno’s neofunctionalization model (Ohno, 1970). Indeed, there are many examples of duplicated genes related to stress response, sensory pathways, transport, and metabolism being fixed under certain environmental conditions (Kondrashov, 2012). Comparative genomics of the yeasts *Saccharomyces cerevisiae* and *Schizosaccharomyces pombe* provided evidence for the role of gene duplication in organismal adaptation (Qian and Zhang, 2014). Similarly, a large-scale genomic analysis of land plants concluded that gene duplication was contributing to the evolution of novel functions, including disease resistance and the production of specific floral structures (Panchy et al., 2016). More recently, it was suggested that hundreds of HSDs are aiding the survival of the Antarctic green alga *Chlamydomonas* sp. UWO241 via gene dosage (Cvetkovska et al., 2018; Zhang et al., 2021a).

The identification of duplicated genes in eukaryotic genomes can be challenging, especially in instances involving functional redundancy, multidomain protein structures, and/or extensive small-scale duplication events (Li et al., 2001; Prince and Pickett, 2002; Li et al., 2003b). There are five broad classes of duplication events in genomes: whole-genome duplication (WGD), tandem duplication, transposon-mediated duplication, segmental duplication, and retroduplication (Panchy et al., 2016). Two methods are typically used to evaluate the paralogous relationships of genes within species: the sequence similarity method and the gene structure method. For example, bioinformatics tools can detect duplicated genes based on their sequence similarity, which is usually measured by looking at three metrics: percentage sequence identity, aligned length, and E-value (Lallemand et al., 2020). There are various tools for rapidly quantifying sequence similarity and alignment length, such as BLAST (Kent, 2002) and DIAMOND (Buchfink et al., 2015). Furthermore, the thresholds of the metrics in the alignment tools are highly reliant on the timescale of paralogs. If the investigated paralogs are ancient, these thresholds have to be lower to remain sensitive. For instance, a BLAST all-against-all protein sequence similarity search usually involves the following thresholds as the cut-off when defining paralogs: ≥30% identity score, E-value cut-off ≤ 1e-5, and an aligned length of ≥150 amino acids (Sander and Schneider, 1991; Maere et al., 2005; Panchy et al., 2016).

More complex similarity-based metrics have also been developed. Rost (1999) and Li et al. (2001) proposed respective formulas based on the threshold curve from homology-derived secondary structures of proteins (HSSP) (Sander and Schneider, 1991). Gene structure can also help reinforce the paralogous relationship of two genes within a species. For instance, the conserved domains and pathways detected by InterPro (Mitchell et al., 2019), Pfam (El-Gebali et al., 2019), and KEGG (Kanehisa and Goto, 2000) can be strong indicators of homology (Lallemand et al., 2020). But they are best used alongside high-quality genome assembly and annotation data, otherwise there is the strong possibility that predicted duplicates will be false positives due to assembly artefacts.

Various bioinformatics tools and software suites have been developed for identifying gene duplications. When choosing tools for identifying duplicate genes, much depends on the biological questions being asked, the genomes being compared, and the bioinformatics skills of the user (Lallemand et al., 2020). GenomeHistory (Conant and Wagner, 2002), for example, is a popular tool, which does not require the user to manually run BLAST searches and also provides information on the synonymous and nonsynonymous substitution rates of duplicate genes. OrthoDB (Zdobnov et al., 2017) and OrthoMCL (Li et al., 2003a) use the graph-based method and Markovian Cluster algorithm to identify in-paralogs within species. Likewise, OrthoFinder (Emms and Kelly, 2015; Emms and Kelly, 2019) can detect orthogroups across species and infer gene duplication events from gene trees. RetrogeneDB was built to identify retrocopies with the criteria that the aligned sequences are at least 150 bp long and have at least 50% amino acid identity and coverage to parental genes (Kabza et al., 2014; Rosikiewicz et al., 2017). It is important to stress, however, that some of these bioinformatics algorithms and associated tools were not specifically designed for detecting duplicates.

There are some previously developed tools and databases for studying gene duplication. The Duplicated Gene Database (DGD), for instance, collected the co-localized and duplicate genes from nine species but has not updated any new species since 2012 (Ouedraogo et al., 2012). In the DGD, two genes were considered as co-localized duplicates when the all-against-all BLAST results were within a 100 gene window and satisfied the previously noted formula (Li et al., 2001). Similarly, the Plant Genome Duplication Database (PGDD) houses gene and genome duplication information (Lee et al., 2012; Lee et al., 2017), but the website no longer appears to be active. More recently, a research group developed a duplication events detection pipeline incorporated with the MCScanX algorithm (Wang et al., 2013) that can detect duplicates in plants derived from whole-genome, tandem, proximal, transposed, or dispersed duplication events (Wang et al., 2011; Qiao et al., 2019) (see the detailed method comparisons in the *Results and Discussion* section).

We recently showed that the nuclear genome of Antarctic green alga *Chlamydomonas* sp. UWO241 harbours hundreds of HSDs, which might be aiding its survival in the cold via gene dosage (Cvetkovska et al., 2018; Zhang et al., 2021a). These HSDs were curated into a filtered gene set whereby each group of duplicates had near-identical protein lengths (within 10 amino acids of each other) and ≥90% pairwise identities (Zhang et al., 2021b). In our analysis of the UWO241 genome, we struggled to find adequate bioinformatics tools to identify, annotate, categorize, and visualize duplicated genes with similar gene structures (i.e., similar Pfam domains and InterPro annotations). Consequently, we designed an easy-to-use, automated, and online software tool called HSDFinder.

The software is catered to identifying highly similar duplicate genes and not necessarily highly divergent duplicates. In other words, HSD finder is best used to find paralogs that are highly similar in sequence and thus likely carry out the same function. Highly similar paralogs likely (but not certainly) arose more recently than more diverged paralogs (i.e., HSDs likely represent more recent duplication events than less similar duplicates). From a functional perspective, HSDs/HS-paralogs probably encode proteins that carry out the same function and thus are more likely to have a role in gene dosage as compared to more divergent duplicates/paralogs.

This software is also designed with a user-friendly interface for parsing BLAST all-against-all protein sequence similarity searches via homology assessment metrics (i.e., amino acid pairwise identity and amino acid length variance); it integrates structural information, including Pfam domains and InterPro annotations, in order to better annotate gene duplicates; it displays the duplicates to be categorized over KEGG pathway schematics; and it offers an online publication-ready heatmap plotting option for visualizing the duplicates across species.

## Materials and Methods

### Requirements and Implementation

HSDFinder can be run on the Apache server through an online web interface designed using HTML and Python scripts (http://hsdfinder.com) or through a local environment (Linux and Python 3) after downloading the software package from GitHub (https://github.com/zx0223winner/HSDFinder). But to run it locally, the pre-installed Python (preferably Python 3) and Linux (e.g., Ubuntu 20.04 LTS) environments are required. Usually, a minimum specification requirement is a machine with two cores and 4 GB of random-access memory (RAM), which should allow HSDs to be identified and visualized within a few minutes. The tested data and external software resources, including links, are listed in the key resources table (Supplementary Table S1). The documentation and tutorials can be found via the GitHub: https://github.com/zx0223winner/HSDFinder.

The software implementation is written in Python 3. There are three groups of custom scripts and platforms: 1) HSDFinder.py, operation.py, and pfam.py filter, which annotate the duplicates from BLAST all-against-all protein similarity search results and protein annotation databases (e.g., Pfam domain); 2) HSD_to_KEGG.py categorizes the duplicates under KEGG pathway functional categories; and 3) Django (3.1.5), a Python-based web platform used to maintain the web server as well as pandas (1.2.2), the software library used for manipulating the data. The full HSDFinder source code can be found in the GitHub repository. Necessary input files include the 12-column BLAST all-against-all protein similarity search output in tab-delimited file and the 13-column InterProScan (Quevillon et al., 2005) search output in a tab-delimited file. The HSD results are summarized in an 8-column tab-delimited file. To create a heatmap of the HSDs under pathway functional categories, the KO accession file with each gene model identifier must be retrieved from the KEGG database internal tools (BlastKOALA or GhostKOALA) (Kanehisa and Goto, 2000; Kanehisa et al., 2016). The result of HSDs under different KEGG functional categories is summarized in an 8-column tab-delimited file. For examples of input and output files, please refer to a published protocol using HSDFinder for analyzing HSDs in seven green algal species (Supplementary Table S1) (Zhang et al., 2021b).

### Software Procedures

**FIGURE 1:**
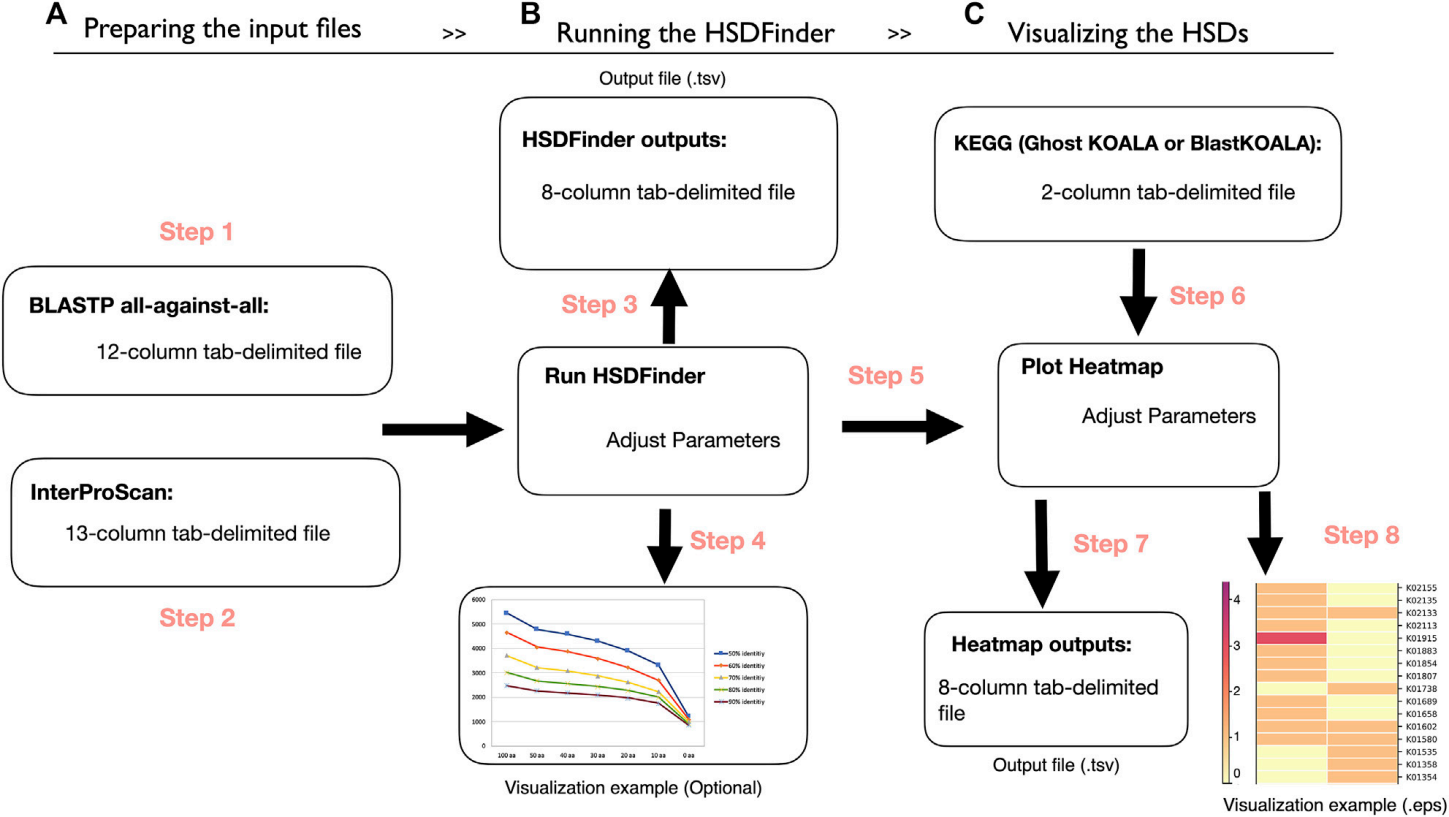
The workflow of HSDFinder. **(A)** Step 1-2: Preparing the protein BLAST all-against-all protein similarity search result file and preparing the InterProScan search result file. **(B)** Step 3-4: Yielding the output of HSDFinder with three personalized options and visualizing the HSDFinder results. **(C)** Step 5-8: Uploading the results of HSDFinder from your respective genomes, uploading a gene list with KO annotation from KEGG database, generating the output files of the online heatmap visualization tool and visualizing the heatmap of HSD levels across species.

Before running HSDFinder, two tab-delimited files created by external tools are needed (Figure 1A). The first is the all-against-all protein sequence BLAST search file (defaulted parameters: E-value cut-off ≤ 1e-5, BLASTP -outfmt 6, -word_size 3, -gapopen 11, -gapextend 1, -max_target_seqs 15). Note, if the species of interest has a large number of gene duplicates, we recommend users enlarge the value of -max_target_seqs. The second is the protein function file acquired from the software InterProScan (defaulted parameters: -f tsv, -dp, -goterms, -pa), which allows protein sequence to be scanned by different protein signature databases (e.g., Pfam domain). Then, the two tab-delimited files can be uploaded to HSDFinder with some personalized options. The default setting of HSDFinder filters HSDs with near-identical protein lengths (within 10 amino acids of each other) and ≥90% pairwise amino acid identities. But users can customize the threshold metrics to optimize their dataset of gene duplicate candidates. The output of HSDFinder is arranged in an 8-column tab-delimited file containing the HSD identifier, gene copy number, and protein signature (e.g., Pfam domain) (Figure 1B). To compare HSDs across different species and visualize HSD results in different KEGG pathway categories, we provide an online heatmap plotting option. Users will need to use the HSD results from the previous steps to employ this feature. Additionally, the file retrieved from the KEGG database documenting the correlation of KEGG Orthology (KO) accession with each gene model identifier will be used to categorize HSDs. Once the two files have been submitted for each species, the HSDs will be displayed in a heatmap (the color for the matrix reflects the number of HSDs across species) and a tab-delimited file under different KEGG functional categories, such as carbohydrate metabolism, energy metabolism, and translation (Figure 1C).

### Software Principles

**FIGURE 2:**
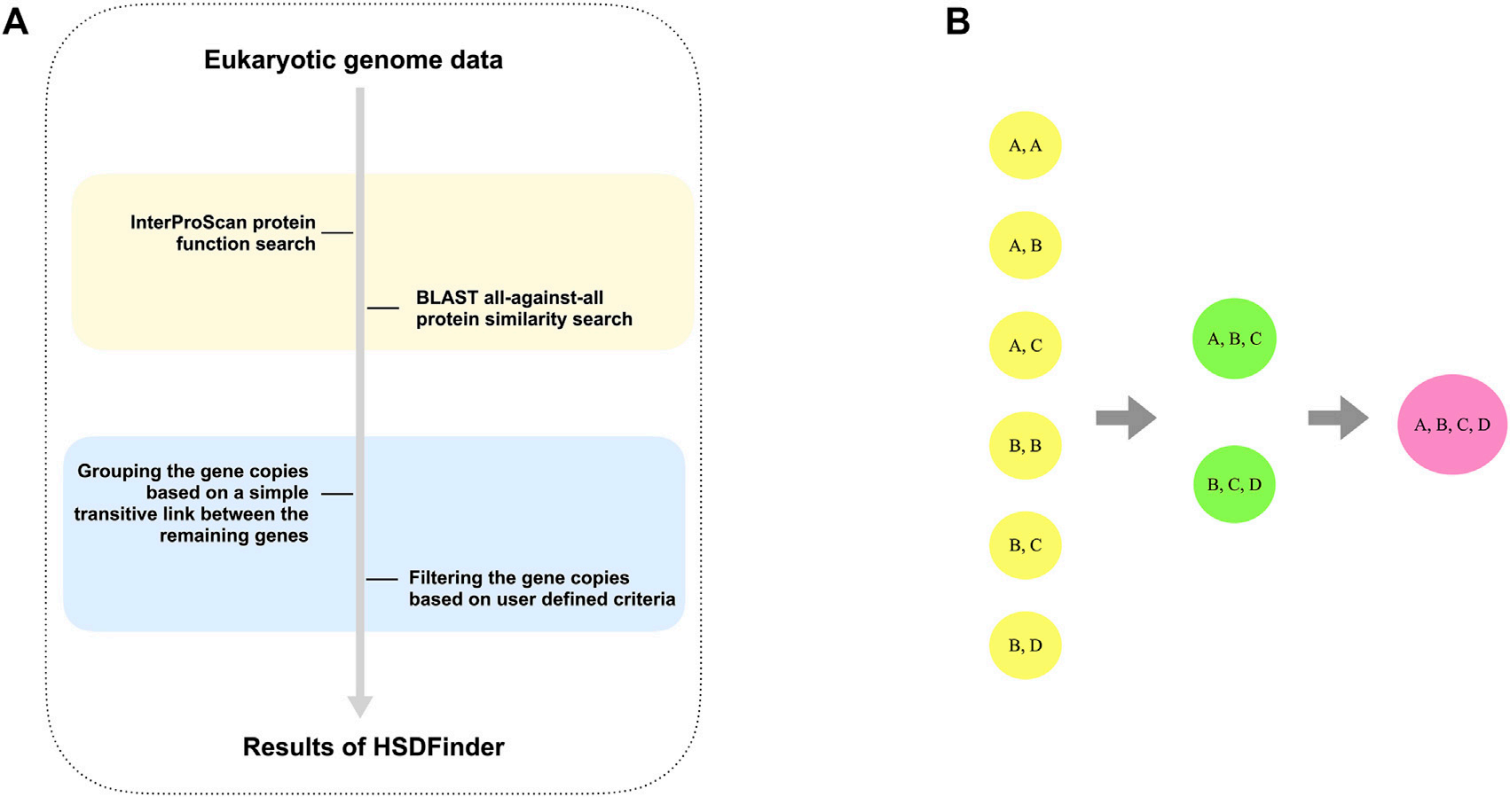
The principle of detecting and grouping the HSDs for eukaryotic genomes. **(A)** The flowchart to parse the BLAST all-against-all protein similarity search result via amino acid pairwise identities and amino acid length variances. **(B)** The principle used to group the satisfied gene copies is based on a simple transitive link between the remaining genes.

HSDFinder is a BLAST-based method, which is designed to parse the BLAST all-against-all protein similarity search result via amino acid pairwise identity and amino acid length variance. By default, HSDFinder filters HSDs with near-identical protein lengths (within 10 amino acids of each other) and ≥90% pairwise amino acid identities. Choosing such a strict cut-off might rule out other genuine duplicates from the list. But based on our past experience with green algal genomes (Zhang et al., 2021a) and validation analyses with some of the best assembled model eukaryotic genomes (discussed in *Results* section), these default thresholds can capture a large number of HSDs. For poorly curated genomes, potential bottlenecks include an increase in the number of hypothetical proteins among predicted HSDs. But since the similarity of duplicated genes within and among genomes can vary significantly, the thresholds can be adjusted (e.g., selecting ≥80% pairwise amino acid identity, still within 10 amino acid length of each other) to acquire more possible HSD candidates (Figure 2A). Similar to the clustering strategy of DGD co-localized genes (Ouedraogo et al., 2012), gene copies in HSD groups were clustered based on the principle of a simple transitive link between the remaining genes: if gene copy A was highly similar to gene copy B and to gene copy C, then gene copies A, B, and C were clustered in the same HSD group, even if gene copies B and C were less similar (Figure 2B). This is also why the amino acid length variances and percent identity thresholds of HSDFinder were set to a default of 10 and 90%, respectively — to increase the prediction accuracy of HSDs, especially for genomes with large numbers of duplicate genes.

## 3 Results and Discussion

### Data Collection

We collected and catalogued HSDs from thirteen nuclear genomes from land plants, animals, and green algae (Table 1). Seven different algal species were selected due to our specific interest in green algal genomics and because of their relatively small genome sizes and gene numbers, which can help decrease the processing time (running time can range from 2–5 min) and central processing unit (CPU) when testing the HSDFinder tool. The other six plant and animal genomes were used to test the performance of HSDFinder. Altogether, we identified 61,656 HSD groups in the thirteen genomes, totaling 191,468 gene copies. The HSD groups with only two, three, and at least four gene copies are 35,776, 11,241, and 14,639, respectively. Compared to the explored green algal genomes, the land plant and animal genomes had higher detected numbers of HSDs, as well as higher ratios of HSDs/Mb and HSDs/total genes (Table 1). For example, the HSDs/Mb values in *A. thaliana*, *O. sativa*, *D. melanogaster* were 61.7, 15.5 and 50.0, respectively, while the largest HSDs/Mb value among selected green algae was 2.5 in *Chlamydomonas eustigma.* This might reflect the diploid nature of the plant and animal genomes, which can yield more gene duplicates via whole-genome duplication events as compared to their haploid green algal counterparts. This can be observed from the results of 3-group HSDs and at least 4-group HSDs in diploid species, which still retain large numbers of HSDs (e.g., 3-group: 1,451 (20% of total) and ≥ 4-group: 995 (13% of total) in *A. thaliana*) compared to the haploid algal species (e.g., 3-group: 26 (10% of total) and ≥ 4-group: 27 (10% of total) in *Chlamydomonas* sp. ICE-L) (Table 1). Note, HSD density is also positively associated with genome size, which tends to be larger in land plants and animals as compared to green algae.

**TABLE 1:** Summary statistics of the predicted HSDs in the thirteen selected eukaryotic genomes.

Domain	Kingdom	Phylum	Class	Order	Species	Genome size (Mb)	No. of considered genes^a^	HSDs #^b^	Gene copies	2-group HSDs #^c^	3-group HSDs #	≥4-group HSDs #	HSDs/Genes	HSDs/Mb	Estimated running time (min)
Eukarya	Plantae	Chlorophyta	Chlorophyceae	Chlamydomonadales	*Chlamydomonas* sp. UWO241	212	16,325	370	1,753	228	43	99	0.023	1.745	3
					*Chlamydomonas reinhardtii*	111	17,741	54	162	34	7	13	0.003	0.486	2
					*Volvox carteri*	131	14,247	124	367	98	12	14	0.009	0.947	2
					*Chlamydomonas eustigma*	110	14,105	276	560	269	6	1	0.020	2.509	2
					*Dunaliella salina*	343	16,697	72	229	56	7	9	0.004	0.210	3
					*Gonium pectorale*	149	16,290	114	325	96	5	13	0.007	0.765	2
					*Chlamydomonas* sp. ICE-L	542	19,870	265	717	212	26	27	0.013	0.489	4
		Streptophyta	Brassicaceae	Brassicales	*Arabidopsis thaliana*	120	48,265	7,404	19,393	4958	1451	995	0.153	61.700	2
			Poaceae	Poales	*Zea mays*	2,198	57,578	9,837	31,477	5941	1677	2219	0.171	4.475	20
					*Oryza sativa*	387	42,580	5,998	16,446	3691	959	1348	0.141	15.499	3
	Animalia	Arthropoda	Insecta	Diptera	*Drosophila melanogaster*	138	30,717	6,894	18,482	4557	1312	1025	0.224	49.957	2
		Chordata	Mammalia	Rodentia	*Mus musculus*	2,690	84,985	15,993	56,734	8153	3014	4826	0.188	5.945	25
					*Rattus norvegicus*	2,632	74,754	14,255	44,823	7483	2722	4050	0.191	5.416	25

a The number of genes listed were retrieved from the source protein FASTA data. To make sure the prediction result can be reproducible, we have not filtered out the organelle genomes if any.

b To best reproduce the work, HSDs were filtered without any manually curation at the uniform parameters: All-against-all protein sequence similarity search using BLASTP (E-value cut-off of ≤1e-5) filtered via the criteria within 10 amino acid length differences and ≥90% amino acid pairwise identities.

c The number of HSDs containing two gene copies.

To explore the functions of detected HSDs, we compared three green algae species all of which had relatively large numbers of HSDs/genes. These algae can survive under different extreme environmental conditions, and include the Antarctic psychrophilic green algae UWO241 (0.021) and *Chlamydomonas* sp. ICE-L (0.013) and the acidophilic species *C. eustigma* (0.020) (Table 1). The identified duplicates are involved in a diversity of cellular pathways, including gene expression, cell growth, membrane transport, and energy metabolism, but also include ribosomal proteins (species: HSDs number/gene copies number; UWO241: 19/42; ICE-L: 41/91; *C. eustigma*: 8/16), histone functional domains (UWO241: 5/99; ICE-L: 8/93; *C. eustigma*: 4/13) (Table 2). Although HSDs for protein translation, DNA packaging, and photosynthesis are particularly prevalent, around 30% of the HSDs are hypothetical proteins without any Pfam domains.

**TABLE 2:** Summary statistics of highly similar duplicate gene (HSDs) functions in selected eukaryotic green algae (*Chlamydomonas* sp. UWO241, *Chlamydomonas* sp. ICE-L, and *Chlamydomonas eustigma*).

Database	Example identifiers^a^	Number of HSDs (%)/Number of gene copies (%)^b^		
		UWO241	ICE-L	*C. eustigma*
Pfam				
Chlorophyll A-B binding protein	PF00504	4 (1%)/25 (2%)	5 (2%)/18 (3%)	3 (1%)/6 (1%)
Ribosomal protein	PF01015; PF01775; PF00828	19 (5%)/42 (3%)	41 (15%)/91(13%)	8 (3%)/16 (3%)
Core histone H2A/H2B/H3/H4	PF00125	5 (1%)/99 (7%)	8 (3%)/93 (13%)	4 (1%)/13 (2%)
Ice-binding protein (DUF3494)	PF11999	8 (2%)/21(2%)	NA	NA
Reverse transcriptases	PF00078	38 (11%)/151(11%)	NA	2 (0.5%)/3 (0.5%)
KEGG				
09101 Carbohydrate metabolism	K13979 (alcohol dehydrogenase)	12 (4%)/89 (7%)	9 (3%)/23(3%)	8 (3%)/16 (3%)
09102 Energy metabolism	K02639 (ferredoxin); K08913 (light-harvesting complex II chlorophyll a/b binding protein 2)	10 (3%)/51 (4%)	10 (4%)/20 (3%)	6 (2%)/15 (3%)
09103 Lipid metabolism	K01054 (acylglycerol lipase)	3 (1%)/15 (1%)	3 (1%)/6 (1%)	6 (2%)/12 (2%)
09122 Translation	K02868 (large subunit ribosomal protein L11e)	27 (8%)/47 (4%)	44 (16%)/97 (16%)	16 (6%)/32 (6%)
Hypothetical Proteins	NA	125 (37%)/357 (27%)	91 (34%)/220 (31%)	88 (32%)/177 (32%)

a Not all identifiers are listed.

b HSDs share ≥90% pairwise amino acid identity and have lengths within 10 amino acid length of each other.

### Performance

**FIGURE 3:**
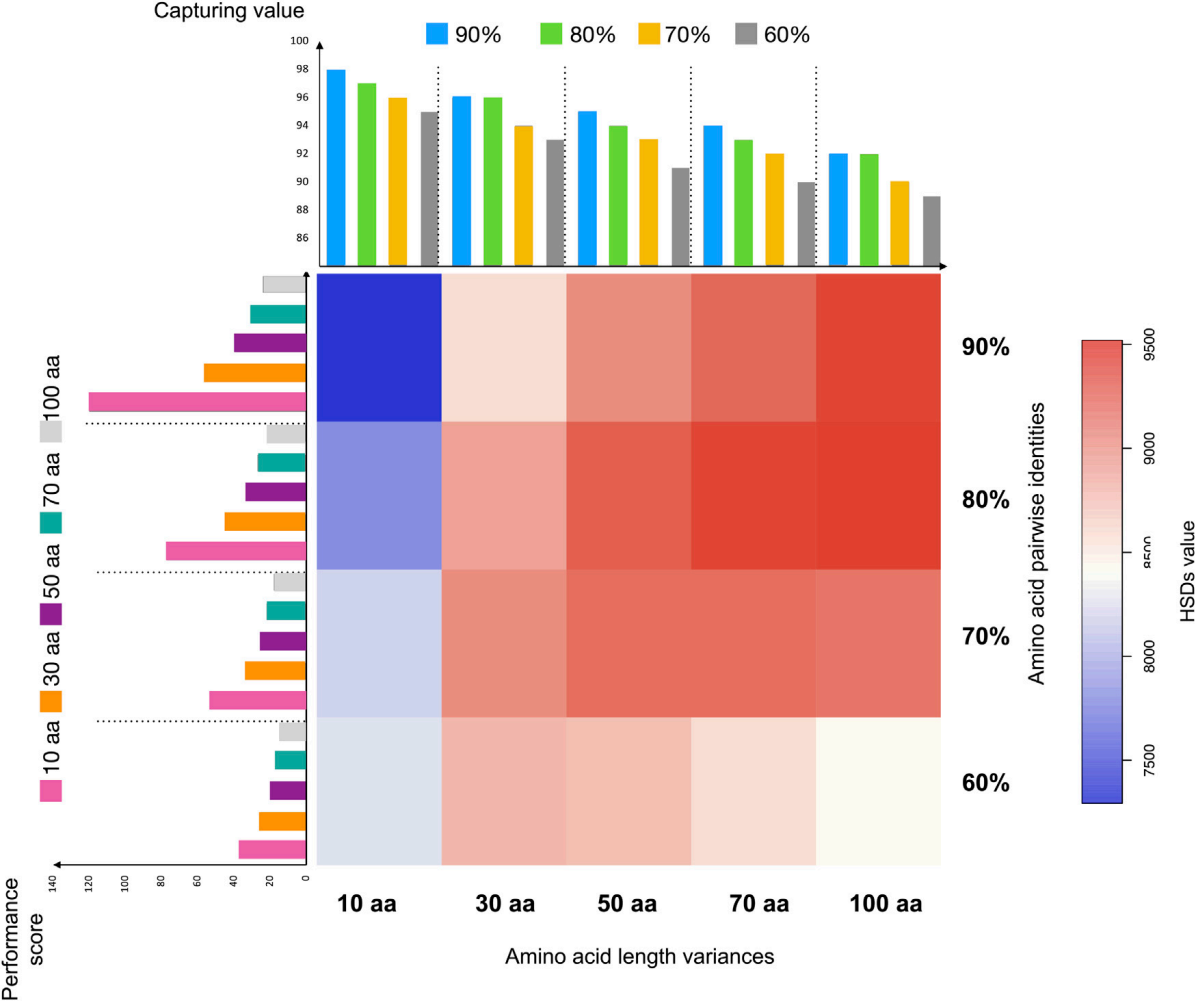
The distribution of duplicates in *Arabidopsis thaliana* detected via different thresholds in HSDFinder. Note: (1) The *X*-axis labelling for heatmap, for example, 10 aa indicates all-against-all protein sequence similarity search using BLASTP (E-value cut-off of ≤1e-5) filtered via the criteria within 10 amino acid length differences; the *Y*-axis labelling for heatmap, for example, 90% indicates ≥90% amino acid pairwise identities; the color matrix indicates the number of true HSDs from the lowest (blue) to highest (red); (2) The *X*-axis labelling for the bar graph on the left-hand side of the heatmap, the color bar indicates the amino acid length threshold, for example, pink for threshold within 10 amino acid length differences (orange for 30 aa, purple for 50 aa, cyan for 70 aa and light grey for 100 aa); the *Y*-axis is the performance score indicating a value to evaluate the performance of detected results. (3) The *X*-axis labelling for the bar graph on the top side of the heatmap, the color bar indicates the amino acid pairwise identity threshold, for example, blue for threshold within 90% amino acid pairwise identities (green for 80%, yellow for 70%, and grey for 60%); the *Y*-axis is the capturing value indicating the capturing ability of predicted HSDs.

To test the performance of HSDFinder, six well-assembled model eukaryotic nuclear genomes were selected, including those of *A. thaliana*, *O. sativa*, *Z. mays*, *D. melanogaster*, *M. musculus*, and *R. norvegicus*. The statistics of HSD candidates in each species via different thresholds are summarized in Table 3 and Supplementary Table S2. The distributions of gene duplicates in each species filtered by various thresholds are presented in Figure 3 and Supplementary Figure S1. Taking the *A. thaliana* genome as an example, an all-against-all protein sequence similarity search using BLASTP (E-value cut-off of ≤1e-5) was filtered via the following criteria: from 10 to 100 amino acid length differences and from ≥60% to ≥90% amino acid pairwise identities (Table 3). The capturing rate of the results and the performance of the BLAST-based tool were evaluated by the following equations:

$$Capturing\;value=\frac{True\;HSDs\;}{True\;HSDs\;+\;Incomplete\;HSDs}\;\times 100\;$$ (1)

$$Performance\;Score=\frac{True\;HSDs\;+\;\left(True\;HSDs\;+\;Incomplete\;HSDs\;-\;Space\right)}{Incomplete\;HSDs\;+\;1}$$

$$\;\;\;\;\;\;\;\;\;\;\;=\frac{2\;\times \;True\;HSDs\;+\;Incomplete\;HSDs\;-\;Space}{Incomplete\;HSDs\;+\;1}$$ (2)

**TABLE 3:** Summary statistics of gene duplicates in *Arabidopsis thaliana* detected via different thresholds in HSDFinder.

Species name	HSD thresholds^a^	Candidate HSDs #	True HSDs #^b^	Space #^c^	Incomplete HSDs #^d^	Capturing value %^e^	Score^f^	2-group gene copies #	3-group gene copies #	≥4-group gene copies #
*Arabidopsis thaliana*	60%_10aa	8647	8245	1584	402	95	37	5,064	1766	1817
	60%_30aa	9447	8797	1831	650	93	25	4,888	1996	2,563
	60%_50aa	9571	8767	1917	804	91	20	4,626	2032	2,913
	60%_70aa	9510	8610	1931	900	90	17	4,416	1997	3,097
	60%_100aa	9472	8434	1921	1038	89	15	4,200	2016	3,256
	70%_10aa	8440	8161	1525	279	96	53	5,251	1,665	1,524
	70%_30aa	9566	9066	1772	500	94	33	5,360	1986	2,220
	70%_50aa	9912	9248	1873	664	93	25	5,239	2082	2,591
	70%_70aa	10030	9254	1896	776	92	22	5,150	2081	2,799
	70%_100aa	10125	9188	1898	937	90	18	4,981	2,155	2,989
	80%_10aa	7970	7787	1427	183	97	77	5,171	1,570	1,229
	80%_30aa	9316	8952	1699	364	96	45	5,587	1920	1809
	80%_50aa	9841	9327	1803	514	94	33	5,596	2081	2,164
	80%_70aa	10095	9458	1840	637	93	27	5,545	2,138	2,412
	80%_100aa	10337	9519	1852	818	92	21	5,472	2,244	2,621
	90%_10aa	7404	7294	1371	110	98	120	4,958	1,451	995
	90%_30aa	8878	8599	1629	279	96	56	5,586	1822	1,470
	90%_50aa	9502	9080	1728	422	95	39	5,722	1993	1787
	90%_70aa	9845	9294	1768	551	94	31	5,745	2084	2016
	90%_100aa	10174	9448	1786	726	92	24	5,738	2,190	2,246

a Gene duplicates were detected via different thresholds in HSDFinder. For example, 60%_10aa indicates all-against-all protein sequence similarity search using BLASTP (E-value cut-off of ≤1e-5) filtered via the criteria within 10 amino acid length differences and ≥60% amino acid pairwise identities.

b True HSDs # are HSD groups satisfying the respective thresholds and the respective gene copies contain same domain(s).

c Space indicates the respective HSDs including the gene copies without any domain(s) (e.g., hypothetical proteins).

d Incomplete HSDs # are HSD groups satisfying the respective thresholds, but the respective gene copies contain different domain(s).

e Capturing % is calculated by Eq. 1, which indicates the capturing ability of predicted HSDs.

f Score is calculated by Eq. 2, which indicates a value to evaluate the performance of detected results.

**FIGURE 4:**
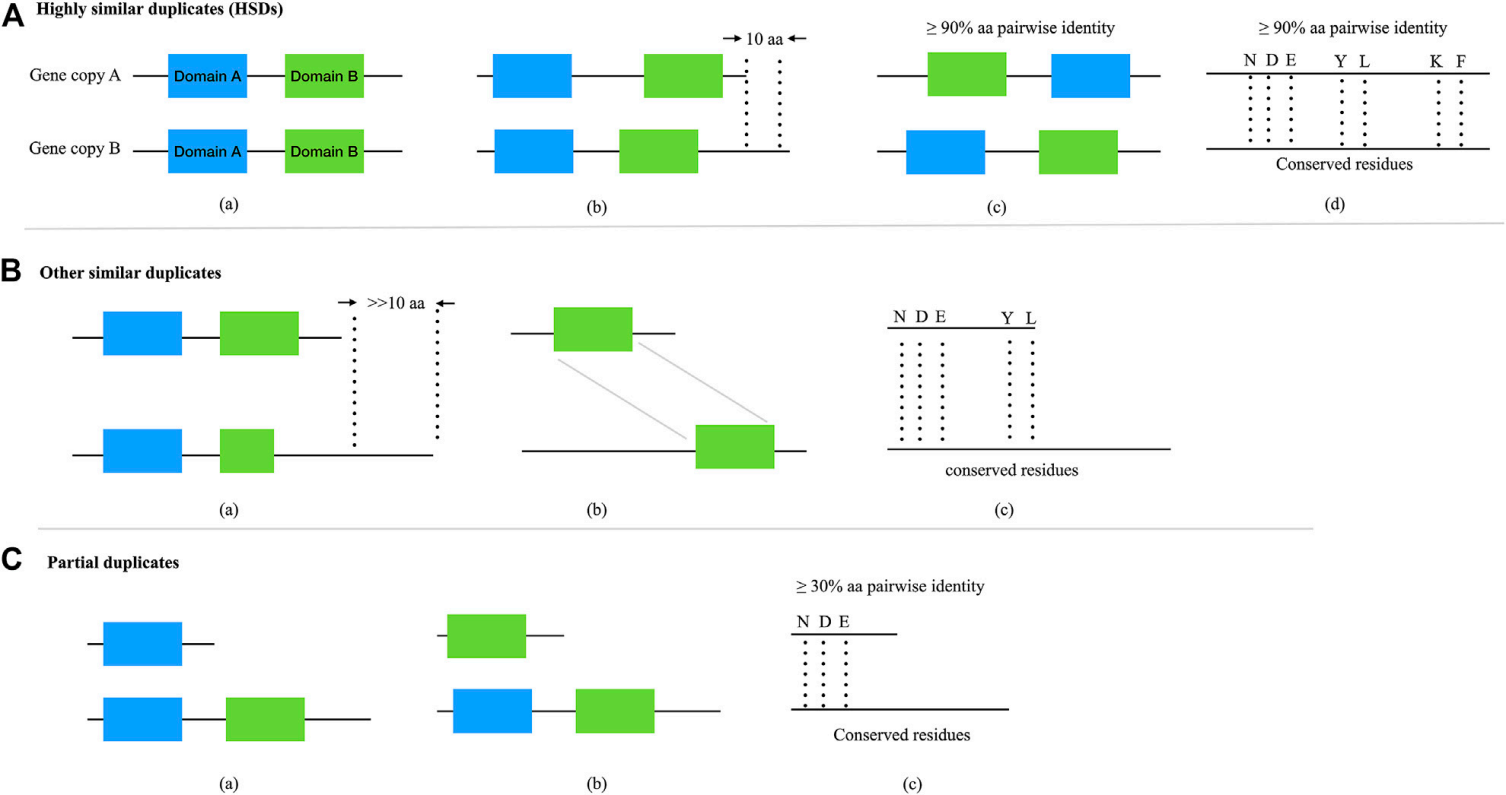
The categories and relationships of complete and partial gene duplicates. **(A)** Complete duplicates with highly similar structures (i.e., HSDs). ^a,b,c^Duplicates with shared domain(s), satisfied with ≥90% protein pairwise identity and ≤10 amino acid length variances; ^b^duplicates share with conserved residues, satisfied with ≥90% protein pairwise identity and ≤10 amino acid length variances. **(B)** Other similar duplicates with highly conserved structure but very different amino acid length. ^a,b,c^Duplicates shared with same domain(s) or residues, satisfied with ≥30% protein pairwise identity (Li et al., 2001) and >>10 amino acid length **(C)** Partial gene duplicates. ^a,b,c^Duplicates partially shared with domain(s) or residues, satisfied with ≥30% protein pairwise identity.

In Table 3, “True HSD #” is the number of HSD groups that satisfy the respective thresholds and for which the respective gene copies contain the same domain(s). “Space” denotes HSDs (including gene copies) without any domain(s) (e.g., hypothetical proteins). “Incomplete HSD #” indicates the number of gene duplicates that satisfy the respective thresholds but for which the associated gene copies contain different domain(s). Note, incomplete HSDs and partial duplicates with differing domain structures could have undergone duplication as well as other evolutionary processes, such as recombination (Long and Langley, 1993; Katju and Lynch, 2003; Zhang et al., 2004). Also, keep in mind that there is the possibility of false positives when identifying gene duplicates. The capturing value Eq. 1 reflects the number of predicted HSDs. As displayed in Figure 3, when keeping the amino acid length at the same level, the capturing value (bar graph at the top) decreases with the amino acid pairwise identity going down. This is true with the amino acid length variance from ≥10 amino acids to ≥100 amino acids. Larger amino acid length variances can result in more partial duplicates (i.e., possible genes copies with different domains), decreasing the capturing rate of predicted HSDs. But loosening the thresholds for amino acid length variance and pairwise identity can increase the sensitivity of prediction (Figure 4). Since a gold standard cut-off is impossible to determine, different metrics will lead to different results (Lallemand et al., 2020). We set the parameters of the default to ≥90% amino acid pairwise identity and 10 amino acid length variances, then refine the possible HSDs candidates from ≥80% amino acid pairwise identity and 10 amino acid length variances. This is a highly conservative sensitivity.

Users of HSDFinder should evaluate the performance of each threshold to best filter the appropriate gene duplicate set. Unfortunately, there are no simulated data available to do the benchmark work (running time, results, false positives, false negatives, etc.). But we introduced a simple equation to roughly evaluate the performance of each metric. For example, in Eq. 2, the numerator is the total of true HSDs plus the HSD groups containing functional domains (Incomplete HSDs + True HSDs -Space). The denominator is the incomplete HSDs plus one, to get rid of zero as a denominator. We designed the software to acquire as many accurate HSD predictions as possible, especially those that contain matching (and complete) domains. Thus, incomplete HSDs results in a penalty score to the denominator, true HSDs and value (Incomplete HSDs + True HSDs -Space) earning a bonus score as the numerator. Taking *A. thaliana* as an example (Figure 3 and Supplementary Figure S1), the performance score reflected the highest value at the threshold of ≥90% amino acid pairwise identity and 10 amino acid length variances, with the second highest value at ≥80% amino acid pairwise identity and 10 amino acid length variances. Similar results were also observed with the other explored genomes (i.e., those of *Z. mays*, *O. sativa*, *D. melanogaster*, *M. musculus* and *R. norvegicus*) (Supplementary Figure S1). Thus, for HSDFinder, we set the default parameters to ≥90% amino acid pairwise identity and 10 amino acid length variances, then refine the possible HSDs candidates from ≥80% amino acid pairwise identity and 10 amino acid length variances.

To validate the performance of these parameters in HSDFinder, we compared the number of duplicated genes predicted by HSDFinder to other previously used methods for *A. thaliana*, *Z. mays*, and *O. sativa* (Table 4)*.* Our detection results gave comparable numbers of nearly identical gene duplicates: 21,516 (HSDFinder) vs 21,622 for *A. thaliana* (Wang et al., 2011; Qiao et al., 2019); 34,581 (HSDFinder) vs 43,000 (Panchy et al., 2016) for *Z. mays*; and 17,989 (HSDFinder) vs 21,461 (Wang et al., 2011; Qiao et al., 2019) for *O. sativa*. Note: we used the most up-to-date assembly versions of the published genomes because HSDFinder is dependent on the existence of high-quality genome assembly and annotation data. For example, in *A. thaliana*, 21,516 and 19,393 gene copies were detected to be highly similar using a ≥80% amino acid pairwise identity and a 10 amino acid length variance and a ≥90% amino acid pairwise identity and a 10 amino acid length variance, respectively. However, 11,937 and 12,761 gene duplicates were collected using BLASTN (all-against-all at ≥40% nucleotide identity) (Blanc and Wolfe, 2004) and BLASTP (all-against-all at ≥30% identity) (Maere et al., 2005). This large discrepancy in the number of duplicates recovered between the two methods is mostly due to the updating of protein annotations in *A. thaliana*. The Arabidopsis Information Resource (TAIR) genome has released ten annotation versions over the past decade.

**TABLE 4:** Comparison of the number of duplicated genes by different methods in model species, such as *Arabidopsis thaliana*, *Oryza sativa* (Rice) and *Zea mays*. Adapted from Lallemand et al. (2020) under the creative commons attribution license.

Species	Type of method detection	No. of median gene count^a^	No. of estimated gene copies	% Estimated Gene Copies^b^	Duplicated gene types	References
*Arabidopsis thaliana*	HSDFinder identified^c^	27,334	21,516	78.7	All paralogous pairs were searched	This article
		27,334	19,393	70.9	All paralogous pairs were searched	This article
	References indicated^d^	22,810	21,622	94.8	WGD, tandem, proximal, DNA based transposed, retrotransposed, and dispersed duplications	Wang et al. (2011); Qiao et al. (2019)
*Zea mays*	HSDFinder^c^	57,578	34,581	60.0	All paralogous pairs were searched	This article
		57,578	31,477	54.7	All paralogous pairs were searched	This article
	References indicated^e^	∼62,000	∼43,000	∼69	All paralogous pairs were searched	Panchy et al. (2016)
*Oryza sativa*	HSDFinder^c^	38,007	17,989	47.3	All paralogous pairs were searched	This article
		38,007	16,446	43.3	All paralogous pairs were searched	This article
	References indicated^d^	27,910	21,461	76.9	WGD, tandem, proximal, DNA based transposed, retrotransposed, and dispersed duplications	Wang et al. (2011), Qiao et al. (2019)

a The number of median gene count were retrieved from each genome assembly version in NCBI.

b These values have been calculated according to the information provided in the corresponding reference article and self-calculated.

c (1) All-against-all protein sequence similarity search using BLASTP (E-value cut-off of ≤1e-5) filtered via the criteria within 10 amino acid length differences and ≥80% amino acid pairwise identities. (2) All-against-all protein sequence similarity search using BLASTP (E-value cut-off of ≤1e-5) filtered via the criteria within 10 amino acid length differences and ≥90% amino acid pairwise identities.

d All-against-all protein sequence similarity search using BLASTP (top five non-self protein matches with E-value of 1e-10 were considered). Genes without hits that met a threshold of E-value 1e-10 were deemed singletons. Pairs of WGD duplicates were downloaded from published lists. Single gene duplications were derived by excluding pairs of WGD duplicates from the population of gene duplications. Tandem duplications were defined as being adjacent to each other on the same chromosome. Proximal duplications were defined as non-tandem genes on the same chromosome with no more than 20 annotated genes between each other. Single gene transposed-duplications were searched for from the remaining single gene duplications using syntenic blocks within and between 10 species to determine the ancestral locus. If the parental copy had more than two exons and the transposed copy was intronless, the pair of duplicates was classified as coming from a retrotransposition. Other cases of single gene-transposed duplications were classified as DNA based transpositions. Dispersed duplications corresponded to the remaining duplications not classified as WGD, tandem, proximal, or transposed duplications.

e A gene is regarded as duplicated if it is significantly similar to another gene in a BLAST search (identity ≥30%, aligned region ≥150 amino acids, E-value cut-off of ≤1e-5).

## 4 Limitations

HSDFinder can identify duplicated genes when the duplicates satisfy the assigned criteria: near-identical protein lengths (within 10 amino acids of each other) and ≥90% pairwise amino acid identities. However, it does not rule out another widespread method for duplication detection based on Best BLAST Mutual Hits (BBMH) (Droc et al., 2006). Unlike the pipeline tool *DupGen_finder* (Wang et al., 2011; Qiao et al., 2019), our software cannot efficiently differentiate duplicates arising from tandem, proximal, dispersed, whole-genome, DNA-based transposon, or retrotransposon duplication events. The limitations of HSDFinder also include the requirement of users to be familiar with the external tools such as the BLAST package, InterProScan, and KEGG’s BlastKOALA and GhostKOALA. But we do provide build-in references for each input file as well as a step-by-step protocol (Zhang et al., 2021b). In our experiences (Zhang et al., 2021a), the default settings of HSDFinder were able to detect a significant proportion of intact duplicated genes, but many fragmented and partial duplicates were missed. Users can employ different metrics to filter for their desired duplicates, and HSDFinder can easily group those duplicates into a list if the genome assembly is of good quality. However, the challenge is to separate complete gene duplicates from divergent partial duplicates. Thus, it is easy to uncover more duplicates via lowering the threshold, but hundreds of partials and divergent paralogs could be generated at the same time. It is our hope in the future to optimize the metrics of sequence similarity (e.g., amino acid sequence similarity and length variance) and protein structure (e.g., Pfam domain) to increase the capturing ability of detecting complete duplicates. The software will also be expanded to consider other types of genomic data, such as prokaryotic and organelle genomes. We will also employ the software on other chlorophyte algae and model eukaryotic genomes. The results will be documented in HSDatabase (http://hsdfinder.com/database/).

## 5 Conclusion

With the decreasing cost of biological analyses (e.g., next-generation sequencing), biologists are dealing with larger amounts of data, and many bioinformatics software analysis suites require considerable knowledge of computer scripting and microprogramming. HSDFinder is designed to fill the demand for custom-made scripts to move from one analysis step to another. It can analyze duplicated genes from genome sequences by integrating the results from InterProScan and KEGG. HSDFinder aims to become a useful platform for the identification and comprehensive analysis of HSDs in eukaryotic genomes. In the future, the software will be improved by taking into account more scientific discoveries in the field of gene duplication, particularly substitution rate analyses and expression levels.

## Data Availability

The datasets of eukaryotes supporting the conclusions of this article are available from JGI (https://phytozome.jgi.doe.gov/pz/portal.html) or NCBI (https://www.ncbi.nlm.nih.gov) database.
