# Supplementary material for: HSDFinder: A BLAST-Based Strategy for Identifying Highly Similar Duplicated Genes in Eukaryotic Genomes
Source: Front Bioinform. 2021 Dec 16;1:803176. doi: 10.3389/fbinf.2021.803176 (PMC9580922; doi:10.3389/fbinf.2021.803176)
Supplement: Supplementary file 1 [file DataSheet1.docx]

Supplementary Material

# Supplementary Figures and Tables

## Supplementary Figures

**Supplementary Figure 1.** The distribution of duplicates in some best assembled species (*Arabidopsis thaliana*, *Zea mays, Oryza sativa, Drosophila melanogaster, Mus musculus and Rattus norvegicus*) via different thresholds in HSDFinder. Note: (1) The X-axis labelling, for example, 50% indicates all-against-all protein sequence similarity search using BLASTP (E-value cut-off of ≤1e-5) filtered via the criteria ≥50% amino acid pairwise identities; the color tabs under the bar graph indicate different amino acid length thresholds, for example, pink for threshold within 10 amino acid length differences (orange for 30 aa, purple for 50 aa, cyan for 70 aa and light grey for 100 aa); (2) Y-axis labelling on the left-hand side, for example, Candidate HSDs (blue bar) indicates the number of highly similar gene duplicates candidates; true HSDs (yellow bar) are duplicates groups satisfying the respective thresholds and the respective gene copies contain same domain(s); space (grey bar) indicates the respective HSD groups including the gene copies without any domain(s) (e.g., hypothetical proteins); (3) Y-axis labelling on the right-hand side, for example, capturing value (blue curve) indicates the capturing ability of predicted HSDs; performance score (green curve) indicates a value to evaluate the performance of detected results.

**

*Arabidopsis thaliana*

**

*Zea mays* (Maize)

**

*Oryza sativa* (Rice)

*Drosophila melanogaster* (Fruit fly)

**

*Mus musculus* (Mouse)

**

*Rattus norvegicus* (Norway Rat)

## Supplementary Tables

**Supplementary Table 1**. **The key resource table includes the genome data and software being used in the HSDFinder tool.** Table was adapted from (Zhang. et al. 2021b) with permission.

| **Data and Software** | **Source *** | **References** |
| --- | --- | --- |
| **Genome data** | | |
| *Chlamydomonas* sp. UWO241 (renamed *Chlamydomonas priscuii*) | GenBank (GCA_016618255) | (Zhang et al., 2021a) |
| *Chlamydomonas reinhardtii* (Green alga) | JGI 5.5 (Phytozome 12.1) | (Merchant et al., 2007) |
| *Volvox carteri* (Green alga) | JGI 2.0 (Phtyzome 12.1) | (Prochnik et al., 2010) |
| *Chlamydomonas eustigma* (Green alga) | GenBank (GCA_002335675.1) | (Hirooka et al., 2017) |
| *Dunaliella salina* (Green alga) | JGI 3.0 (Phytozome 12.1) | (Polle et al., 2017) |
| *Gonium pectorale* (Green alga) | GenBank (GCA_001584585.1) | (Hanschen et al., 2016) |
| *Chlamydomonas* sp. ICE-L (Green alga) | GenBank (GCA_013435795.1) | (Zhang et al., 2020) |
| *Arabidopsis thaliana* | GenBank (GCA_000001735.2) | (Rhee et al., 2003) |
| *Zea mays* (Maize) | GenBank (GCA_902167145.1) | (Maier et al., 1995, Schnable et al., 2009) |
| *Oryza sativa* (Rice) | GenBank (GCF_001433935.1) | (Kawahara et al., 2013, Sakai et al., 2013) |
| *Drosophila melanogaster* (Fruit fly) | GenBank (GCA_000001215.4) | (Adams et al., 2000) |
| *Mus musculus* (Mouse) | GenBank (GCA_000001635.9) | (Waterston and Pachter, 2002) |
| *Rattus norvegicus* (Norway Rat) | GenBank (GCF_015227675.2) | (Gibbs and Pachter, 2004) |
| **Software and algorithms** | | |
| BLAST v2.2.26 | ftp://ftp.ncbi.nlm.nih.gov/blast/executables/blast+/LATEST/ | (Kent, 2002) |
| InterProScan v4.7 | http://www.ebi.ac.uk/interpro/download/ | (Quevillon et al., 2005) |
| BlastKOALA or GhostKOALA | https://www.kegg.jp | (Kanehisa and Goto, 2000, Kanehisa et al., 2016) |
| HSDFinder v1.0 | http://hsdfinder.com; https://github.com/zx0223winner/HSDFinder | This article |
| Python 3 | https://www.python.org/downloads/ | NA |
| Django v3.1.5 | https://www.djangoproject.com/download/ | NA |
| pandas v1.2.2 | https://pandas.pydata.org | NA |

*Accession numbers are from the Joint Genome Institute (JGI) and US National Center for Biotechnology Information (NCBI) GenBank assembly accession.

**Supplementary Table 2**. Summary statistics of duplicates predicted in multiple species (*Zea mays, Oryza sativa, Drosophila melanogaster, Mus musculus and Rattus norvegicus*) via different thresholds of HSDFinder.

| **Species name** | **HSD categories** | **Candidate HSDs #** | **True HSDs #** | **Space #** | **Incomplete HSDs#** | **Capturing %** | **Score** | **2-group gene copies #** | **3-group gene copies#** | **>=4-group gene copies#** |
| --- | --- | --- | --- | --- | --- | --- | --- | --- | --- | --- |
| *Zea mays* (Maize) | 50%_10aa | 10320 | 9601 | 2256 | 719 | 93 | 24.53 | 5187 | 1882 | 3251 |
|  | 50%_30aa | 9558 | 8616 | 2266 | 942 | 90 | 16.87 | 4142 | 1745 | 3671 |
|  | 50%_50aa | 9076 | 8063 | 2216 | 1013 | 88 | 14.72 | 3629 | 1665 | 3782 |
|  | 50%_70aa | 8788 | 7715 | 2174 | 1073 | 87 | 13.34 | 3401 | 1587 | 3800 |
|  | 50%_100aa | 8515 | 7372 | 2147 | 1143 | 86 | 12.01 | 3227 | 1517 | 3771 |
|  | 60%_10aa | 10696 | 10114 | 2236 | 582 | 94 | 31.86 | 5698 | 1967 | 3031 |
|  | 60%_30aa | 10532 | 9653 | 2310 | 879 | 91 | 20.31 | 4943 | 1927 | 3662 |
|  | 60%_50aa | 10184 | 9205 | 2269 | 979 | 90 | 17.47 | 4462 | 1882 | 3840 |
|  | 60%_70aa | 9965 | 8900 | 2249 | 1065 | 89 | 15.59 | 4223 | 1847 | 3895 |
|  | 60%_100aa | 9804 | 8641 | 2230 | 1163 | 88 | 13.93 | 4060 | 1815 | 3929 |
|  | 70%_10aa | 10839 | 10370 | 2215 | 469 | 95 | 40.41 | 6096 | 1982 | 2761 |
|  | 70%_30aa | 10839 | 10370 | 2215 | 469 | 95 | 40.41 | 6096 | 1982 | 2761 |
|  | 70%_50aa | 10839 | 10370 | 2215 | 469 | 95 | 40.41 | 6096 | 1982 | 2761 |
|  | 70%_70aa | 10839 | 10370 | 2215 | 469 | 95 | 40.41 | 6096 | 1982 | 2761 |
|  | 70%_100aa | 10699 | 9599 | 2274 | 1100 | 89 | 16.37 | 4870 | 1960 | 3869 |
|  | 80%_10aa | 10579 | 10204 | 2111 | 375 | 96 | 49.66 | 6211 | 1863 | 2505 |
|  | 80%_30aa | 10971 | 10351 | 2207 | 620 | 94 | 30.78 | 5909 | 1923 | 3139 |
|  | 80%_50aa | 10997 | 10239 | 2221 | 758 | 93 | 25.05 | 5626 | 1982 | 3389 |
|  | 80%_70aa | 10968 | 10111 | 2222 | 857 | 92 | 21.98 | 5462 | 1988 | 3518 |
|  | 80%_100aa | 10989 | 9995 | 2232 | 994 | 90 | 18.85 | 5361 | 1990 | 3638 |
|  | 90%_10aa | 9837 | 9575 | 1939 | 262 | 97 | 66.44 | 5941 | 1677 | 2219 |
|  | 90%_30aa | 10330 | 9851 | 2042 | 479 | 95 | 37.79 | 5812 | 1779 | 2739 |
|  | 90%_50aa | 10465 | 9858 | 2084 | 607 | 94 | 30.00 | 5664 | 1841 | 2960 |
|  | 90%_70aa | 10503 | 9810 | 2102 | 693 | 93 | 26.24 | 5552 | 1860 | 3091 |
|  | 90%_100aa | 10558 | 9745 | 2117 | 813 | 92 | 22.34 | 5480 | 1857 | 3221 |
| *Oryza sativa* (Rice) | 50%_10aa | 7682 | 7239 | 1492 | 443 | 94 | 30.25 | 4024 | 1423 | 2235 |
|  | 50%_30aa | 8175 | 7519 | 1697 | 656 | 91 | 21.30 | 3683 | 1538 | 2954 |
|  | 50%_50aa | 8186 | 7403 | 1706 | 783 | 90 | 17.71 | 3433 | 1491 | 3262 |
|  | 50%_70aa | 8190 | 7303 | 1727 | 887 | 89 | 15.50 | 3307 | 1513 | 3370 |
|  | 50%_100aa | 8192 | 7189 | 1760 | 1003 | 87 | 13.57 | 3204 | 1510 | 3478 |
|  | 60%_10aa | 7436 | 7126 | 1438 | 310 | 95 | 42.20 | 4155 | 1360 | 1921 |
|  | 60%_30aa | 8228 | 7756 | 1642 | 472 | 94 | 30.32 | 4159 | 1584 | 2485 |
|  | 60%_50aa | 8438 | 7855 | 1662 | 583 | 93 | 25.05 | 4055 | 1594 | 2789 |
|  | 60%_70aa | 8516 | 7863 | 1690 | 653 | 92 | 22.46 | 3975 | 1626 | 2915 |
|  | 60%_100aa | 8614 | 7864 | 1726 | 750 | 91 | 19.64 | 3928 | 1653 | 3033 |
|  | 70%_10aa | 7019 | 6817 | 1365 | 202 | 97 | 61.43 | 4139 | 1220 | 1660 |
|  | 70%_30aa | 7874 | 7531 | 1552 | 343 | 95 | 40.27 | 4336 | 1472 | 2066 |
|  | 70%_50aa | 8153 | 7729 | 1585 | 424 | 94 | 33.64 | 4309 | 1534 | 2310 |
|  | 70%_70aa | 8300 | 7810 | 1614 | 490 | 94 | 29.52 | 4298 | 1587 | 2415 |
|  | 70%_100aa | 8448 | 7888 | 1646 | 560 | 93 | 26.19 | 4295 | 1633 | 2520 |
|  | 80%_10aa | 6499 | 6382 | 1302 | 117 | 98 | 98.13 | 3945 | 1096 | 1458 |
|  | 80%_30aa | 7258 | 7051 | 1472 | 207 | 97 | 61.72 | 4190 | 1320 | 1748 |
|  | 80%_50aa | 7581 | 7300 | 1504 | 281 | 96 | 47.44 | 4270 | 1394 | 1917 |
|  | 80%_70aa | 7755 | 7417 | 1529 | 338 | 95 | 40.24 | 4300 | 1446 | 2009 |
|  | 80%_100aa | 7923 | 7528 | 1565 | 395 | 95 | 35.07 | 4335 | 1506 | 2082 |
|  | 90%_10aa | 5998 | 5932 | 1239 | 66 | 98 | 159.57 | 3691 | 959 | 1348 |
|  | 90%_30aa | 6675 | 6537 | 1384 | 138 | 97 | 85.09 | 3958 | 1145 | 1572 |
|  | 90%_50aa | 6971 | 6782 | 1409 | 189 | 97 | 64.97 | 4054 | 1207 | 1710 |
|  | 90%_70aa | 7138 | 6900 | 1439 | 238 | 96 | 52.72 | 4094 | 1265 | 1779 |
|  | 90%_100aa | 7308 | 7011 | 1470 | 297 | 95 | 43.12 | 4149 | 1320 | 1839 |
| *Drosophila melanogaster* (Fruit fly) | 50%_10aa | 6915 | 6811 | 1366 | 104 | 98 | 117.71 | 4360 | 1345 | 1210 |
|  | 50%_30aa | 7135 | 6989 | 1476 | 146 | 97 | 86.04 | 4107 | 1478 | 1550 |
|  | 50%_50aa | 7132 | 6951 | 1505 | 181 | 97 | 69.11 | 3952 | 1479 | 1701 |
|  | 50%_70aa | 7102 | 6896 | 1513 | 206 | 97 | 60.31 | 3813 | 1483 | 1806 |
|  | 50%_100aa | 7046 | 6794 | 1522 | 252 | 96 | 48.69 | 3669 | 1469 | 1908 |
|  | 60%_10aa | 6930 | 6855 | 1356 | 75 | 98 | 163.54 | 4448 | 1355 | 1127 |
|  | 60%_30aa | 7238 | 7131 | 1460 | 107 | 98 | 119.53 | 4269 | 1490 | 1479 |
|  | 60%_50aa | 7272 | 7132 | 1491 | 140 | 98 | 91.58 | 4144 | 1504 | 1624 |
|  | 60%_70aa | 7266 | 7103 | 1500 | 163 | 97 | 78.47 | 4025 | 1508 | 1733 |
|  | 60%_100aa | 7225 | 7018 | 1507 | 207 | 97 | 61.23 | 3901 | 1488 | 1836 |
|  | 70%_10aa | 6916 | 6864 | 1351 | 52 | 99 | 234.51 | 4501 | 1340 | 1075 |
|  | 70%_30aa | 7258 | 7173 | 1453 | 85 | 98 | 150.91 | 4369 | 1486 | 1403 |
|  | 70%_50aa | 7323 | 7203 | 1486 | 120 | 98 | 107.77 | 4265 | 1499 | 1559 |
|  | 70%_70aa | 7336 | 7192 | 1495 | 144 | 98 | 89.88 | 4161 | 1517 | 1658 |
|  | 70%_100aa | 7313 | 7125 | 1502 | 188 | 97 | 68.44 | 4041 | 1506 | 1766 |
|  | 80%_10aa | 6901 | 6861 | 1350 | 40 | 99 | 302.73 | 4530 | 1322 | 1049 |
|  | 80%_30aa | 7260 | 7190 | 1449 | 70 | 99 | 183.11 | 4413 | 1488 | 1359 |
|  | 80%_50aa | 7326 | 7223 | 1482 | 103 | 98 | 125.64 | 4311 | 1500 | 1515 |
|  | 80%_70aa | 7349 | 7222 | 1490 | 127 | 98 | 102.20 | 4209 | 1529 | 1611 |
|  | 80%_100aa | 7328 | 7157 | 1497 | 171 | 97 | 75.51 | 4090 | 1519 | 1719 |
|  | 90%_10aa | 6894 | 6856 | 1355 | 38 | 99 | 317.82 | 4557 | 1312 | 1025 |
|  | 90%_30aa | 7243 | 7180 | 1443 | 63 | 99 | 202.81 | 4435 | 1482 | 1326 |
|  | 90%_50aa | 7318 | 7225 | 1473 | 93 | 98 | 139.04 | 4344 | 1502 | 1472 |
|  | 90%_70aa | 7341 | 7225 | 1483 | 116 | 98 | 111.82 | 4250 | 1525 | 1566 |
|  | 90%_100aa | 7321 | 7160 | 1490 | 161 | 97 | 80.19 | 4126 | 1516 | 1679 |
| *Mus musculus* (Mouse) | 50%_10aa | 15120 | 14116 | 1810 | 1004 | 93 | 27.29 | 7114 | 2855 | 5151 |
|  | 50%_30aa | 13417 | 12162 | 1760 | 1255 | 91 | 18.96 | 5299 | 2410 | 5708 |
|  | 50%_50aa | 12304 | 10868 | 1615 | 1436 | 88 | 15.00 | 4253 | 2117 | 5934 |
|  | 50%_70aa | 11690 | 10110 | 1561 | 1580 | 86 | 12.80 | 3741 | 1956 | 5993 |
|  | 50%_100aa | 11068 | 9317 | 1485 | 1751 | 84 | 10.79 | 3287 | 1815 | 5966 |
|  | 60%_10aa | 15589 | 14858 | 1817 | 731 | 95 | 39.11 | 7551 | 2935 | 5103 |
|  | 60%_30aa | 14394 | 13355 | 1782 | 1039 | 93 | 24.97 | 5897 | 2618 | 5879 |
|  | 60%_50aa | 13425 | 12159 | 1645 | 1266 | 91 | 18.89 | 4823 | 2360 | 6242 |
|  | 60%_70aa | 12928 | 11488 | 1601 | 1440 | 89 | 15.83 | 4331 | 2217 | 6380 |
|  | 60%_100aa | 12448 | 10766 | 1534 | 1682 | 86 | 12.88 | 3879 | 2099 | 6470 |
|  | 70%_10aa | 15850 | 15288 | 1817 | 562 | 96 | 52.08 | 7813 | 2999 | 5038 |
|  | 70%_30aa | 14977 | 14100 | 1785 | 877 | 94 | 31.08 | 6300 | 2755 | 5922 |
|  | 70%_50aa | 14194 | 13064 | 1661 | 1130 | 92 | 22.63 | 5237 | 2607 | 6350 |
|  | 70%_70aa | 13814 | 12481 | 1616 | 1333 | 90 | 18.50 | 4773 | 2492 | 6549 |
|  | 70%_100aa | 13409 | 11808 | 1549 | 1601 | 88 | 14.77 | 4341 | 2374 | 6694 |
|  | 80%_10aa | 16068 | 15602 | 1812 | 466 | 97 | 63.94 | 8086 | 3023 | 4959 |
|  | 80%_30aa | 15463 | 14679 | 1798 | 784 | 95 | 36.11 | 6700 | 2848 | 5915 |
|  | 80%_50aa | 14808 | 13729 | 1683 | 1079 | 93 | 24.86 | 5683 | 2714 | 6411 |
|  | 80%_70aa | 14448 | 13167 | 1638 | 1281 | 91 | 20.26 | 5196 | 2608 | 6644 |
|  | 80%_100aa | 14112 | 12528 | 1578 | 1584 | 89 | 15.81 | 4750 | 2512 | 6850 |
|  | 90%_10aa | 15993 | 15594 | 1786 | 399 | 98 | 74.50 | 8153 | 3014 | 4826 |
|  | 90%_30aa | 15652 | 14947 | 1808 | 705 | 95 | 40.78 | 6968 | 2904 | 5780 |
|  | 90%_50aa | 15106 | 14109 | 1707 | 997 | 93 | 27.56 | 6020 | 2817 | 6269 |
|  | 90%_70aa | 14770 | 13565 | 1661 | 1205 | 92 | 22.12 | 5510 | 2717 | 6543 |
|  | 90%_100aa | 14459 | 12927 | 1598 | 1532 | 89 | 16.82 | 5068 | 2599 | 6792 |
| *Rattus norvegicus* (Norway Rat) | 50%_10aa | 14492 | 13920 | 1539 | 572 | 96 | 46.90 | 6999 | 2814 | 4679 |
|  | 50%_30aa | 14161 | 13111 | 1597 | 1050 | 92 | 24.43 | 5424 | 2520 | 6217 |
|  | 50%_50aa | 13515 | 12095 | 1580 | 1420 | 89 | 16.91 | 4415 | 2303 | 6797 |
|  | 50%_70aa | 13147 | 11495 | 1561 | 1652 | 87 | 13.96 | 3905 | 2140 | 7102 |
|  | 50%_100aa | 12728 | 10775 | 1505 | 1953 | 84 | 11.26 | 3384 | 1977 | 7367 |
|  | 60%_10aa | 14474 | 14018 | 1526 | 456 | 96 | 59.01 | 7153 | 2796 | 4525 |
|  | 60%_30aa | 14418 | 13569 | 1583 | 849 | 94 | 31.06 | 5774 | 2620 | 6024 |
|  | 60%_50aa | 14013 | 12805 | 1574 | 1208 | 91 | 20.88 | 4898 | 2476 | 6639 |
|  | 60%_70aa | 13771 | 12345 | 1559 | 1426 | 89 | 17.21 | 4422 | 2371 | 6978 |
|  | 60%_100aa | 13436 | 11695 | 1501 | 1741 | 87 | 13.56 | 3912 | 2243 | 7281 |
|  | 70%_10aa | 14465 | 14092 | 1518 | 373 | 97 | 72.30 | 7301 | 2783 | 4381 |
|  | 70%_30aa | 14614 | 13879 | 1589 | 735 | 94 | 36.55 | 6074 | 2700 | 5840 |
|  | 70%_50aa | 14366 | 13273 | 1585 | 1093 | 92 | 23.82 | 5277 | 2612 | 6477 |
|  | 70%_70aa | 14231 | 12904 | 1573 | 1327 | 90 | 19.25 | 4849 | 2536 | 6846 |
|  | 70%_100aa | 13982 | 12341 | 1520 | 1641 | 88 | 15.11 | 4354 | 2442 | 7186 |
|  | 80%_10aa | 14478 | 14149 | 1507 | 329 | 97 | 82.18 | 7452 | 2776 | 4250 |
|  | 80%_30aa | 14770 | 14105 | 1594 | 665 | 95 | 40.96 | 6333 | 2751 | 5686 |
|  | 80%_50aa | 14612 | 13598 | 1588 | 1014 | 93 | 26.23 | 5588 | 2689 | 6335 |
|  | 80%_70aa | 14534 | 13284 | 1575 | 1250 | 91 | 20.98 | 5184 | 2622 | 6728 |
|  | 80%_100aa | 14327 | 12757 | 1526 | 1570 | 89 | 16.27 | 4681 | 2557 | 7089 |
|  | 90%_10aa | 14255 | 13987 | 1477 | 268 | 98 | 99.50 | 7483 | 2722 | 4050 |
|  | 90%_30aa | 14750 | 14162 | 1590 | 588 | 96 | 46.39 | 6547 | 2776 | 5427 |
|  | 90%_50aa | 14697 | 13767 | 1593 | 930 | 93 | 28.86 | 5876 | 2734 | 6087 |
|  | 90%_70aa | 14646 | 13493 | 1581 | 1153 | 92 | 23.01 | 5498 | 2686 | 6462 |
|  | 90%_100aa | 14466 | 12992 | 1527 | 1474 | 89 | 17.58 | 4996 | 2633 | 6837 |
